# Association analysis of *ADRB3*:rs4994 with urodynamic outcome, six months after a single intra-detrusor injection of botulinum toxin, in women with overactive bladder

**DOI:** 10.1007/s43440-024-00647-9

**Published:** 2024-09-11

**Authors:** Sylwester Ciećwież, Klaudyna Lewandowska, Aleksandra Szylińska, Agnieszka Boroń, Dariusz Kotlęga, Jacek Kociszewski, Agnieszka Brodowska, Jeremy S.C. Clark, Andrzej Ciechanowicz

**Affiliations:** 1 Department of Gynecology, Endocrinology and Gynecological Oncology, Pomeranian Medical University, Szczecin, Poland; 2 Department of Clinical and Molecular Biochemistry, Pomeranian Medical University, Szczecin, Poland; 3 Department of Cardiac Surgery, Pomeranian Medical University, Szczecin, Poland; 4 Department of Pharmacology and Toxicology, University of Zielona Góra, Zielona Góra, Poland; 5 Department of Gynecology, Evangelisches Krankenhaus Hagen, Hagen, Germany

**Keywords:** Beta-3 adrenoreceptor, Botulinum neurotoxin-A, Gene polymorphism, Overactive bladder, urodynamic assessment

## Abstract

**Background:**

Intra-detrusor injection of botulinum neurotoxin type A (BoNT/A) is recommended as a possible treatment for patients with overactive bladder (OAB) in whom first-line therapies have failed. The c.190T > C (rs4994) polymorphism in the gene encoding the beta-3 adrenergic receptor (*ADRB3*) has been suggested to be associated with predisposition to OAB or with response to OAB treatment via a cholinergic muscarinic receptor antagonist. This prospective study aimed to use a urodynamic parameter-based assessment of response, six months after a single intra-detrusor injection of BoNT/A in female OAB patients, to elucidate possible association with the *ADRB3* polymorphism.

**Methods:**

The study group consisted of 138 consecutive, Polish, adult, female OAB patients. Urodynamic parameters were recorded before injection of BoNT/A and at six months after administration. *ADRB3*:rs4994 variants were identified by the sequencing of genomic DNA extracted from buccal swabs.

**Results:**

Apart from baseline, and relative, increase in Maximum Cystometric Capacity (MCC) six months after BoNT/A injection, no significant differences were found in urodynamic parameters between reference TT homozygotes and women with at least one C allele.

**Conclusions:**

Our results do not exclude that *ADRB3*:rs4994 variants are associated with a positive urodynamic test-based response to intra-detrusor injection of BoNT/A in females with OAB.

## Introduction

According to the International Continence Society (ICS), overactive bladder syndrome (OAB) is defined as urinary urgency, usually with urinary frequency and nocturia, with urgency urinary incontinence or without [1, 2]. The syndrome is heterogeneous and comprises both cases with a neurological background (neurogenic OAB), with multiple sclerosis as a primary cause, and cases in which the underlying mechanism cannot be identified (idiopathic OAB) [3]. Despite many years of research, the mechanism of OAB is yet to be understood; however, a large body of evidence from both clinical and experimental studies suggests that the risk factors for OAB include aging, obesity, obstruction of the bladder’s outlet, psychological stress, and genetic susceptibility, to mention a few [4]. In 2021 Rashid et al. revealed that OAB in Pakistani women was associated with diabetes mellitus, income, parity, and urinary tract infections [5]. In addition, Barone et al. reported worsening of lower urinary tract symptoms in patients who suffered anxiety and stress from the COVID-19 pandemic [6].

The theory of genetic susceptibility to OAB is supported primarily by the familial occurrence of the disease [7–9] and the identification of genetic polymorphisms predisposing to urinary incontinence in genome-wide association studies [10–12].

The authors of a review paper published in 2022 presented some evidence which suggested that idiopathic OAB may be associated with autonomic dysfunction, i.e., an imbalance between the parasympathetic nervous system (mainly involved in emptying the bladder) and sympathetic drive (responsible for the storage of urine). The latter’s contribution to urine storage by relaxing the detrusor muscle involves the activation of G-protein-coupled beta-3 adrenergic receptors [13]. Human beta-3 adrenergic receptor is encoded by the *ADRB3* gene located at chromosome 8p11.23 [14–16]. The *ADRB3* c.190T > C (rs4994) transition results in a change of tryptophan (W) to arginine (R) at position 64 of the beta-3 adrenergic receptor protein (p.Trp64Arg = p.W64R). The p.64Arg variant is implicated in biochemical dysfunction of the beta-3 adrenergic receptor through downregulation of its activation [16–18], but available data on p.64Arg hypofunctionality originate primarily from in vitro studies [16].

The results of two published meta-analyses suggest that a link may exist between the p.Trp64Arg polymorphism and OAB susceptibility [15, 19]. However, as highlighted by Michel [20], in only two out of four studies included in those meta-analyses [16, 17, 21, 22] was the frequency of the p.64Arg allele in OAB patients higher than in healthy controls.

It is also worth noting that a one-year administration of oxybutynin, a cholinergic muscarinic receptor antagonist, in a small sample of Turkish children with OAB, was highly effective in subjects homozygous for the *ADRB3* T allele (*n* = 30), but not in TC heterozygotes (*n* = 4), as shown by both a lesser severity of micturition disorders and higher bladder volume on urodynamic assessment [23].

In line with recent guidelines, intravesical injection of botulinum toxin type A (BoNT/A) is recommended as second- or third-line treatment in patients with detrusor overactivity, in patients which have not responded adequately to a first-line therapy [24]. Treatment with BoNT/A may constitute an alternative to neural stimulation in OAB patients [25]. However, as shown by Abrar et al., some patients may respond sub-optimally to BoNT/A treatment and/or experience adverse events, such as micturition disorders requiring clean, intermittent, self-catheterization or urinary tract infections [26].

The results of our previous study, published in 2022, suggested no relationship between the *ADRB3*:rs4994 polymorphism and the response of Polish female OAB patients to a single intra-detrusor BoNT/A injection, three months post-injection (response measured as absolute and relative changes in OAB symptom frequency or with scores for ICIQ-LUTS-QoL (International Consultation Incontinence Questionnaire-Lower Urinary Tract Symptoms-Quality of Life) and ICIQ-OAB (International Consultation on Incontinence Questionnaire-Overactive Bladder) questionnaires [27]. In a systematic review published in 2020, Abrar et al. [26] identified male sex, concomitant diseases, older age, tobacco smoking, baseline leakage episodes, and some urodynamic parameters including bladder outlet obstruction index, high Maximum Detrusor Pressure (Pdet-max) before treatment [28], and poor bladder compliance [29], as predictors of poor response to BoNT/A or adverse events after its intra-detrusor administration. However, as emphasized by the authors of the systematic review, the quality of the source studies was relatively low, which limited the value of the evidence [26].

Therefore, the aim of the present prospective study was to analyze possible association between the *ADRB3*:rs4994 polymorphism and urodynamic changes, as well as patient-perceived changes, in Polish, female OAB patients, six months after a single intra-detrusor injection of BoNT/A.

## Materials and methods

### Female patients with overactive bladder

All women provided written informed consent to participate in the study, and the protocol of the study was approved by the Local Bioethics Committee at the Pomeranian University in Szczecin (decision no. KB-0012/125/17; 16th November 2017). The study group consisted of 138 consecutive, female, patients (ages between 22 and 86 years) diagnosed with OAB using the ICIQ-OAB questionnaire, at the Department of Gynecology, Endocrinology and Gynecological Oncology, Pomeranian Medical University in Szczecin, Western Pomerania, Poland. Female patients suffering from neurogenic OAB (nOAB, *n* = 52), or idiopathic OAB (iOAB, *n* = 86) were diagnosed. All patients were of Polish nationality and European descent and lived in the Polish Western Pomerania region. The eligibility criteria for the study were the same as described previously [27]. Briefly, inclusion criteria were: detrusor overactivity confirmed by urodynamics testing, and intolerance to or ineffective pharmacological treatment of OAB. Exclusion criteria included: previous use of BoNT/A; mixed urinary incontinence; presence of urinary tract infection; bladder stones; bladder cancer; urinary retention; and/or previous urogynecological surgeries.

Determination of baseline characteristics of the patients before BoNT/A injection followed the same protocol as described previously [27] and included: age; body height; body mass; body mass index (BMI), calculated as body mass (kg)/(height (m))^2^; number of pregnancies; number of deliveries; and number of cesarean sections. BoNT/A (100 units, Botox^®^, Allergan Inc., Irvine, CA, USA) was diluted in 10 ml of normal saline (0.9% NaCl). All injections were administered under general anesthesia by the same urogynecologist (S.C.). Using a rigid cystoscope and a needle (injeTak^®^, Laborie, Portsmouth, NH, USA), BoNT/A was injected into the detrusor muscle at 20 sites, 5 sites per point with points distributed approximately evenly above the bladder, including the dome but not the trigone. Before the BoNT/A injection, a buccal swab was collected from each patient to extract genomic DNA.

Before the BoNT/A injection and six months thereafter, urodynamic parameters were determined according to ICS standards in all OAB patients, following the protocol described previously [30]. In brief, urodynamic testing was performed by the same investigator (S.C) after confirming a normal result from urinalysis. Two types of Menfis bioMedica catheters (9 Fr and 12 Fr) were used in the study. One 9 Fr catheter was inserted into the urinary bladder to perform flow cystometry and measure flow pressure. A second 12 Fr catheter was inserted into the rectum to measure intra-abdominal pressure. Every female patient had the urodynamic examination performed in the gynecological position. During the urodynamic examination, 0.9% physiological saline was administered to the urinary bladder at a flow rate of 50–75 ml/min. During the examination, a cough stress test and Valsalva maneuver were performed after administering the first 50 ml of fluid and repeated after every additional 100 ml. The micturition phase started after maximum filling of the bladder. The patient voided to a container placed on a scale, and the uroflow curve and residual urine volume were determined. All urodynamic parameters used were defined according to Rosier et al. [31], Drake et al. [32], and Yao et al. [33], and Post-Void Residual (PVR), First Sensation of Bladder Filling (FSBF), Maximum Flow Rate (Qmax), Average Flow Rate (Qave), Detrusor Pressure at Maximum Flow (Pdet at Qmax), and Maximum Detrusor Pressure (Pdet-max) were determined using the Libra + system (Medical Measurement Systems B.V., Amsterdam, Netherlands).

Information about frequency, nocturia, urgency, and urgency incontinence was obtained from all patients twice: before the BoNT/A injection and six months thereafter. The study participants were asked to complete the ICIQ-OAB and ICIQ-LUTS-QoL questionnaires at both study time points. Raw overall scores for parts A and B of the ICIQ-OAB ranged between 0 and 16 points and 0 to 40 points, respectively, whereas raw overall scores for parts A and B of the ICIQ-LUTS-QoL ranged between 0 and 76 points and 0 to 190 points, respectively.

Absolute (Δ _0−6_) and relative (∆_%_) differences were calculated as the values determined at six months post-injection minus the pretreatment values (divided by the pretreatment values for ∆_%_); for urodynamic parameters, OAB symptom scores, and questionnaire scores.

### Genotyping

Extraction of genomic DNA, amplification of a 440 base-pair (bp) *ADRB3* sequence including the polymorphism of interest (rs4994), and sequencing, were conducted analogously to our previous study [27]. Diploid SNP bases, e.g., homozygote [T]; [T], heterozygote [T]; [C] and homozygote [C]; [C] are abbreviated in this article as TT, TC (= CT), and CC, respectively.

### Statistical analyses and presentation

Whether quantitative variables were normally distributed was tested using Shapiro-Wilk tests. Most quantitative variables were not normally distributed and the summary statistics were presented as medians, minima, and maxima. Whether between-OAB type-group differences were significant in the values of quantitative variables was determined using Mann-Whitney tests. Whether between-genotype-group differences were significant in the values of quantitative variables was determined using logistic regression with the adjustement for OAB type. Whether *ADRB3*:rs4994 genotype frequencies diverged from Hardy-Weinberg Equilibrium (HWE), and tests involving categorical variables, were analyzed using Pearson’s chi-squared (*χ*^2^) tests. The threshold for statistical significance was set at *p* < 0.05. All analyses were carried out with Dell Statistica software, version 13 (Dell Inc. 2016, TX, USA, software.dell.com, accessed on 2nd February 2024).

## Results

There were 124 TT homozygotes (89.8%), 11 TC heterozygotes (8.0%), and 3 CC homozygotes (2.2%) in the studied group of 138 female OAB patients. The frequency of the minor *ADRB3*:c.190 C allele was 6.1% (17 out of 276 alleles). In the subgroup of women with iOAB, there were 77 TT homozygotes (89.6%), 7 TC heterozygotes (8.1%), and 2 CC homozygotes (2.3%), and the frequency of the minor *ADRB3*:c.190 C allele was 6.4% (11 out of 172 alleles). In the subgroup of women with nOAB, there were 47 TT homozygotes (90.4%), 4 TC heterozygotes (7.7%), and one CC homozygote (1.9%), and the frequency of the minor *ADRB3*:c.190 C allele was 5.8% (6 out of 104 alleles). There were no significant differences between iOAB and nOAB patients in the distribution of *ADRB3* genotypes (*χ*^2^ statistic = 0.035, *p* = 0.983) or alleles (*χ*^2^ statistic = 0.044, *p* = 0.834), respectively.

The distributions of *ADRB3*:rs4994 genotypes in the entire group (iOAB + nOAB) and in iOAB or in nOAB subgroups were not consistent with HWE (*χ*^2^ statistic = 13.3, *p* = 0.001; *χ*^2^ statistic = 8.82, *p* = 0.003 or *χ*^2^ statistic = 4.45, *p* = 0.035, respectively).

Women with iOAB also did not differ significantly from those with nOAB in terms of age, body mass and body height, BMI, number of pregnancies, deliveries, and cesarean sections (Table 1). The iOAB and nOAB groups did not differ significantly in terms of urodynamic parameters, other than pretreatment PVR and Pd at Qmax. The pretreatment values of both of these parameters were significantly higher in women with nOAB than in those with iOAB (Table 2). In addition, no significant differences were also noted between the iOAB and nOAB subgroups in terms of frequency, nocturia, urgency, and urgency incontinence, as well as in terms of the scores for ICIQ-OAB part A, ICIQ-OAB part B, ICIQ-LUTS-QoL part A and ICIQ-LUTS-QoL part B before the treatment and six months after BoNT/A injection. The subgroups also did not differ significantly with regard to the absolute or relative differences in all symptom and questionnaire scores (Tables 3 and 4, respectively).

**Table 1:** Basic characteristics of female patients with idiopathic (iOAB) or neurogenic (nOAB) overactive bladder syndrome

Variable	All patients	iOAB	nOAB	U	*p*
	(*n* = 138)	(*n* = 86)	(*n* = 52)		
Age [years]	60.0 (49.0:69.0)	60.5 (51.0:70.0)	56.0 (41.0:68.0)	1841.5	0.083
Body height [cm]	164 (160:167)	164 (158:166)	164 (162:167)	1879.5	0.118
Body mass [kg]	72.0 (65.0:80.0)	72.5 (65.0:80.0)	72.0 (65.0:80.0)	2156.0	0.727
BMI [kg/m^2^]	26.7 (24.2:30.4)	27.5 (24.2:30.5)	26.3 (24.2:29.0)	2029.0	0.364
Pregnancies, n	2 (1:3)	2 (2:3)	2 (1:3)	2125.0	0.627
Deliveries, n	2 (1:2)	2 (1:2)	2 (1:2)	2229.5	0.979
Cesarean sections, n	0 (0:0)	0 (0:0)	0 (0:0)	2131.0	0.646

iOAB, idiopathic overactive bladder; nOAB, neurogenic overactive bladder; BMI, body mass index

Data presented as medians (lower quartile: upper quartile). The “U” (U statistics) and “p” values are for Mann-Whitney tests with a null hypothesis of no difference between iOAB and nOAB

**Table 2:** Urodynamic assessment of female patients, before and six months after intra-detrusor injection of botulinum neurotoxin type A, with idiopathic (iOAB) or neurogenic (nOAB) overactive bladder syndrome

Variables	Time Code	All patients	iOAB	nOAB	U	p
		(*n* = 138)	(*n* = 86)	(*n* = 52)		
MCC [ml]	0	210 (160:244)	215 (165–248)	205 (152–238)	1980.0	0.262
	6	356 (312:367)	356 (345:367)	345 (301:367)	1905.0	0.177
	∆_0–6_	-135 (-193:-93)	-136 (-201:-94)	-131 (-182:84)	2126.0	0.630
	∆_%_	-61 (-119:-38)	-64 (-121:-38)	-58 (-113:-38)	2195.5	0.860
PVR [ml]	0	3 (0:33)	0 (0:21)	23 (0:44)	1602.5	0.005
	6	8 (0:23)	3 (0:10)	8 (0:23)	1883.5	0.148
	∆_0–6_	0 (-4:18)	0 (-4:9)	6 (-3.5:30)	1863.5	0.102
	∆_%_	54 (-2:100)	51 (-10:100)	67 (32:100)	584.0	0.317
FSBF [ml]	0	52 (28:98)	51 (24:98)	55 (33:97)	2085.5	0.510
	6	137 (134:157)	137 (134:157)	156 (134:157)	2171.5	0.866
	∆_0–6_	-92 (-118:-35)	-93 (-119:-35)	-76 (-110:-32)	2091.5	0.527
	∆_%_	-179 (-389:-34)	-180 (-509:-37)	-170 (-278:-29)	2055.0	0.428
Qmax [ml/s]	0	17 (16:19)	18 (16:19)	17 (16:18)	1982.5	0.266
	6	14 (13:17)	14 (13:18)	14 (13:17)	1915.0	0.191
	∆_0–6_	2 (0:5)	2 (0:5)	2 (0:4)	2123.5	0.623
	∆_%_	13 (0:26)	14 (0:28)	13 (0:24)	2147.5	0.699
Qave [ml/s]	0	8 (8:9)	8 (8:9)	8 (8:9)	2103.5	0.562
	6	8 (8:11)	9 (8:11)	8 (8:10)	1929.5	0.214
	∆_0–6_	0 (-2:1)	0 (-2:1)	0 (-1:1)	2168.0	0.767
	∆_%_	0 (-22:11)	0 (-22:11)	0 (-13:11)	2153.5	0.719
Pdet at Qmax [cm H_2_O]	0	37 (35:46)	36 (34:43)	39 (36:47)	1706.0	0.020
	6	20 (19:21)	20 (19:20	20 (19:23)	1803.5	0.072
	∆_0–6_	17 (15:26)	17 (15:25)	18 (16:28)	1942.5	0.198
	∆_%_	47 (43:57)	46 (42:57)	49 (43:57)	2068.5	0.463
Pdet-max [cm H_2_O]	0	51 (38:72)	50 (37:68)	56 (42:83)	1812.0	0.063
	6	27 (26:28)	27 (26:28)	27 (26:34)	1994.5	0.340
	∆_0–6_	24 (11:42)	23 (10:41)	26 (17:48)	1937.0	0.190
	∆_%_	48 (28:59)	46 (27:56)	50 (33:60)	2002.5	0.306

Time codes: 0 = before injection; 6 = 6 months after injection; ∆_0–6_ = absolute difference (∆_%_ = relative difference): value at 6 months after injection minus value before injection;

iOAB, idiopathic overactive bladder; nOAB, neurogenic overactive bladder; MCC, Maximum Cystometric Capacity; PVR, Post Void Residual; FSBF, First Sensation of Bladder Filling; Qmax, Maximum Flow Rate; Qave, Average Flow Rate; Pdet at Qmax, Detrusor Pressure at Maximum Flow; Pdet-max, Maximum Detrusor Pressure

Data presented as medians (lower quartile: upper quartile). The “U” (U statistics) and “p” values are for Mann-Whitney tests with a null hypothesis of no difference between iOAB and nOAB

**Table 3:** Overactive bladder symptoms in female patients, before and six months after intra-detrusor injection of botulinum neurotoxin type A, with idiopathic (iOAB) or neurogenic (nOAB) overactive bladder syndrome

Symptoms	Time Code	All patients	iOAB	nOAB	U	*p*
		(*n* = 138)	(*n* = 86)	(*n* = 52)		
Frequency	0	3 (2:4)	3 (2:3)	3 (2:4)	2006.0	0.313
(range: 0 to 4)	6	1 (0:2)	1 (1:2)	1 (0:2)	2100.0	0.911
	∆_0–6_	1 (1:2)	1 (1:2)	1 (1:2)	2048.5	0.594
	∆_%_	67 (25:100)	50 (33:75)	67 (25:100)	2005.5	0.537
Nocturia	0	3 (2:4)	3 (2:4)	3 (2:4)	2187.0	0.831
(range: 0 to 4)	6	1 (1:2)	1 (1:2)	1 (1:3)	2044.0	0.714
	∆_0–6_	1 (0.5:2)	1 (1:2)	1 (0:2)	1990.0	0.426
	∆_%_	50 (12:67)	50 (33:67)	50 (0:75)	2051.0	0.602
Urgency	0	4 (3:4)	4 (3:4)	4 (3:4)	2215.0	0.930
(range: 0 to 4)	6	2 (1:2)	1 (1:2)	2 (1:3)	1942.0	0.406
	∆_0–6_	2 (1:2)	2 (1:2)	2 (1:2)	2113.5	0.810
	∆_%_	50 (33:67)	50 (33:67)	50 (25:75)	2070.5	0.747
Urgency incontinence	0	3 (2:3)	3 (2:3)	3 (2:3)	2157.0	0.730
(range: 0 to 4)	6	1 (1:2)	1 (1:2)	1 (1:2)	1987.0	0.531
	∆_0–6_	1 (0:2)	1 (0:2)	1 (0:2)	2046.0	0.587
	∆_%_	50 (33:67)	50 (33:67)	50 (25:67)	1828.5	0.602

Time codes: 0 = before injection; 6 = 6 months after injection; ∆_0–6_ = absolute difference (∆_%_ = relative difference): value at 6 months after injection minus value before injection

iOAB, idiopathic overactive bladder; nOAB, neurogenic overactive bladder

Data presented as medians (lower quartile: upper quartile). The “U” (U statistics) and “p” values are for Mann-Whitney tests with a null hypothesis of no difference between iOAB and nOAB

**Table 4:** ICIQ-OAB and ICIQ-LUTSqol questionnaire responses from female patients, before and six months after intra-detrusor injection of botulinum neurotoxin type A, with idiopathic (iOAB) or neurogenic (nOAB) overactive bladder syndrome

Symptoms	Time Code	All patients	iOAB	nOAB	U	*p*
		(*n* = 138)	(*n* = 86)	(*n* = 52)		
ICIQ-OAB, part A	0	11 (10:13)	11 (10:13)	12 (10:13)	2100.5	0.553
(range: 0 to 16)	6	5 (3:7)	5 (4:7)	5 (3:8)	2182.0	0.814
	∆_0–6_	6 (4:8)	6 (4:8)	5 (3:8)	2160.0	0.740
	∆_%_	54 (31:67)	54 (36:67)	55 (28:71)	2197.0	0.866
ICIQ-OAB, part B	0	36 (32:40)	36 (31:40)	37 (32:40)	2091.5	0.527
(range: 0 to 40)	6	13 (5:20)	12 (5:22)	13 (6:25)	2009.5	0.321
	∆_0–6_	21 (9:30)	22 (10:31)	20 (8:28)	1986.0	0.273
	∆_%_	62 (32:87)	65 (37:87)	59 (23:82)	2009.5	0.321
ICIQ-LUTS-QoL, part A	0	57 (47:65)	57 (48:56)	55 (46:64)	2115.0	0.596
(range: 0 to 76)	6	35 (25:49)	33 (24:47)	38 (26:52)	2044.0	0.400
	∆_0–6_	20 (5:30)	25 (6:30)	16 (3:26)	1910.5	0.153
	∆_%_	37 (10:54)	39 (11:56)	31 (7:51)	1973.0	0.249
ICIQ-LUTS-QoL, part B	0	139 (105:164)	141 (111:165)	136 (99:163)	2044.0	0.400
(range: 0 to 190)	6	38 (8:11)	32 (8:107)	61 (10:127)	1940.5	0.195
	∆_0–6_	85 (23:124)	91 (31:127)	59 (13:109)	1874.0	0.112
	∆_%_	63 (19:93)	71 (24:94)	55 (12:92)	1922.5	0.169

Time codes: 0 = before injection; 6 = 6 months after injection; ∆_0–6_ = absolute difference (∆_%_ = relative difference): value at 6 months after injection minus value before injection

iOAB, idiopathic overactive bladder; nOAB, neurogenic overactive bladder;

ICIQ-OAB, International Consultation on Incontinence Questionnaire-Overactive Bladder; ICIQ-LUTS-QoL, International Consultation Incontinence Questionnaire-Lower Urinary Tract Symptoms-Quality of Life

Data presented as medians (lower quartile: upper quartile). The “U” (U statistics) and “p” values are for Mann-Whitney tests with a null hypothesis of no difference between iOAB and nOAB

To analyze the relationship between the response to BoNT/A treatment and *ADRB3* polymorphism, the two subgroups of patients with the different OAB types were pooled into a single group. Given the low frequency of CC homozygotes, in further analyses, the three women with *ADRB3*:rs4994 CC genotype were also pooled with the 11 TC heterozygotes. After this, a logistic regression analysis was conducted within the iOAB + nOAB group, with a correction for OAB type.

Other than for MCC, TT homozygotes did not differ from women with at least one C allele (group TC + CC) in terms of urodynamic parameters. The pretreatment MCC values were significantly lower, but the values of relative MCC increase recorded six months after BoNT/A administration were significantly higher, in women with at least one C allele compared with in women homozygous for the reference allele (TT) (Table 5).

**Table 5:** Urodynamic assessment of female patients, before and six months after intra-detrusor injection of botulinum neurotoxin type A, with regard to *ADRB3*: rs4994 genotype

Variables	Time Code	ADRB3:rs4994 genotype		W	*p*
		TT (*n* = 124)	TC + CC (*n* = 11 + 3)		
MCC [ml]	0	216 (163:246)	169 (143:210)	4.100	0.043
	6	356 (312:367)	345 (345:367)	0.290	0.590
	∆_0–6_	-128 (-178:-87)	-161 (-213:-133)	1.651	0.199
	∆_%_	-58 (-106:-37)	-89 (-149:-57)	4.814	0.028
PVR [ml]	0	1 (0:33)	7 (0:44)	0.028	0.867
	6	3 (0:21)	19 (0:23)	2.369	0.124
	∆_0–6_	0 (-4:18)	0 (-4:21)	0.367	0.545
	∆_%_	62 (-2:100)	50 (4:67)		0.843
FSBF [ml]	0	54 (28:98)	43 (28:60)	0.514	0.474
	6	137 (134:157)	156 (134:165)	0.573	0.449
	∆_0–6_	-85 (-118:-35)	-104 (-115:-74)	1.193	0.275
	∆_%_	-176 (-389:-32)	-235 (-388:-82)	0.228	0.633
Qmax [ml/s]	0	17 (16:19)	17 (15:18)	0.514	0.214
	6	14 (13:17)	14 (13:16)	0.184	0.668
	∆_0–6_	2 (0:5)	2 (1:5)	0.803	0.370
	∆_%_	13 (0:26)	13 (7:28)	0.451	0.502
Qave [ml/s]	0	8 (8:9)	8 (8:9)	1.159	0.282
	6	8 (8:11)	8 (8:9)	0.073	0.787
	∆_0–6_	0 (-2:1)	0 (-1:1)	0.945	0.331
	∆_%_	0 (-22:11)	0 (-13:11)	0.589	0.443
Pdet at Qmax [cm H_2_O]	0	37 (35:46)	36 (34:52)	0.397	0.529
	6	20 (19:21)	20 (19:20)	0.785	0.376
	∆_0–6_	17 (15:26)	17 (15:34)	0.137	0.712
	∆_%_	47 (43:57)	47 (44:59)	2.205	0.138
Pdet-max [cm H_2_O]	0	51 (39:70)	41 (34:80)	0.002	0.968
	6	27 (26:28)	27 (26:34)	0.045	0.832
	∆_0–6_	24 (12:42)	15 (8:45)	0.004	0.949
	∆_%_	48 (28:59)	37 (24:59)	0.240	0.624

Time codes: 0 = before injection; 6 = 6 months after injection; ∆_0–6_ = absolute difference (∆_%_ = relative difference): value at 6 months after injection minus value before injection;

*ADRB3*, gene encoding beta-3 adrenergic receptor; MCC, Maximum Cystometric Capacity; PVR, Post Void Residual; FSBF, First Sensation of Bladder Filling; Qmax, Maximum Flow Rate; Qave, Average Flow Rate; Pdet at Qmax, Detrusor Pressure at Maximum Flow; Pdet-max, Maximum Detrusor Pressure

The “W” (Wald statistics) and “p” values are for OAB type-adjusted logistic regression analyses with a null hypothesis of no difference between TT homozygotes and patients with at least one C allele for the *ADRB3*:rs4994 (c.190T > C) polymorphism

The TT homozygotes did not differ significantly from the TC + CC group in terms of frequency, nocturia, urgency and urgency incontinence, as well as in terms of the scores for ICIQ-OAB part A, ICIQ-OAB part B, ICIQ-LUTS-QoL part A and ICIQ- LUTS-QoL part B before treatment and six months after BoNT/A injection. Similarly, no statistically-significant between-group differences were observed for absolute or relative differences in all symptom and questionnaire scores (Tables 6 and 7, respectively).

**Table 6:** Overactive bladder symptoms in female patients, before and six months after intra-detrusor injection of botulinum neurotoxin type A, in regard to *ADRB3*: rs4994 genotype

Symptoms	Time Code	ADRB3:rs4994 genotype		W	*p*
		TT (*n* = 124)	TC + CC (*n* = 11 + 3)		
Frequency	0	3 (2:4)	2 (2:4)	0.412	0.521
(range: 0 to 4)	6	1 (0:2)	1 (0:1)	0.700	0.403
	∆_0–6_	1 (1:2)	1 (1:3)	0.008	0.930
	∆_%_	67 (25:100)	58 (50:100)	0.005	0.942
Nocturia	0	3 (2:4)	3 (3:4)	0.044	0.834
(range: 0 to 4)	6	1 (1:2)	1 (1:2)	0.157	0.692
	∆_0–6_	1 (1:2)	1 (0:2)	0.031	0.861
	∆_%_	50 (25:67)	42 (0:67)	0.167	0.683
Urgency	0	4 (3:4)	4 (3:4)	0.677	0.411
(range: 0 to 4)	6	2 (1:2)	2 (1:3)	0.250	0.617
	∆_0–6_	2 (1:2)	2 (1:3)	0.016	0.899
	∆_%_	50 (33:67)	58 (25:75)	0.044	0.834
Urgency incontinence	0	3 (2:3)	3 (2:4)	1.454	0.228
(range: 0 to 4)	6	1 (1:2)	1 (1:3)	1.204	0.272
	∆_0–6_	1 (0:2)	1(1:2)	0.023	0.881
	∆_%_	50 (33:67)	50 (25:67)	0.036	0.849

Time codes: 0 = before injection; 6 = 6 months after injection; ∆_0–6_ = absolute difference (∆_%_ = relative difference): value at 6 months after injection minus value before injection;

*ADRB3*, gene encoding beta-3 adrenergic receptor

The “W” (Wald statistics) and “p” values are for OAB type-adjusted logistic regression analyses with a null hypothesis of no difference between TT homozygotes and patients with at least one C allele for the *ADRB3*:rs4994 (c.190T > C) polymorphism

**Table 7:** ICIQ-OAB and ICIQ-LUTSqol questionnaire scores in female patients with overactive bladder, before and six months after intra-detrusor injection of botulinum neurotoxin type A, in regard to *ADRB3*: rs4994 genotype

Symptoms	Time Code	ADRB3:rs4994 genotype		W	*p*
		TT (*n* = 124)	TC + CC (*n* = 11 + 3)		
ICIQ-OAB, part A	0	11 (10:13)	12 (10:14)	0.165	0.684
(range: 0 to 16)	6	5 (4:7)	4 (3:8)	0.056	0.813
	∆_0–6_	6 (4:8)	6 (3:7)	0.002	0.960
	∆_%_	54 (33:67)	55 (27:67)	0.026	0.872
ICIQ-OAB, part B	0	37 (31:40)	35 (34:39)	0.167	0.682
(range: 0 to 40)	6	12 (5:22)	12 (4:32)	0.087	0.768
	∆_0–6_	21 (10:30)	26 (8:31)	0.005	0.946
	∆_%_	61 (37:87)	69 (20:89)	0.105	0.746
ICIQ-LUTS-QoL, part A	0	57 (48:66)	56 (44:62)	0.364	0.546
(range: 0 to 76)	6	35 (26:50)	35 (20:41	0.203	0.653
	∆_0–6_	19 (5:30)	24 (6:32)	0.000	0.994
	∆_%_	36 (10:53)	41 (14:56)	0.002	0.967
ICIQ-LUTS-QoL, part B	0	139 (108:165)	131 (87:161)	0.479	0.489
(range: 0 to 190)	6	38 (10:115)	46 (5:73)	0.263	0.608
	∆_0–6_	81 (24:124)	96 (16:135)	0.001	0.974
	∆_%_	63 (19:93)	68 (18:96)	0.003	0.954

Time codes: 0 = before injection; 6 = 6 months after injection; ∆_0–6_ = absolute difference (∆_%_ = relative difference): value at 6 months after injection minus value before injection;

*ADRB3*, gene encoding beta-3 adrenergic receptor; ICIQ-OAB, International Consultation on Incontinence Questionnaire-Overactive Bladder; ICIQ-LUTS-QoL, International Consultation Incontinence Questionnaire-Lower Urinary Tract Symptoms-Quality of Life

The “W” (Wald statistics) and “p” values are for OAB type-adjusted logistic regression analyses with a null hypothesis of no difference between TT homozygotes and patients with at least one C allele for the *ADRB3*:rs4994 (c.190T > C) polymorphism

## Discussion

To the best of our knowledge, this is the first study analyzing the association of *ADRB3*:rs4994 polymorphism with the urodynamic response and patient-perceived response measured six months after intra-detrusor BoNT/A injection in female OAB patients. The results showed no significant differences between patients with the reference genotype (TT) and those with at least one C allele (TC or CC) in terms of analyzed urodynamic parameters, i.e., Maximum Cystometric Capacity (MCC), Post Void Residual (PVR), First Sensation of Bladder Filling (FSBF), Maximum Flow Rate (Qmax), Average Flow Rate (Qave), Detrusor Pressure at Maximum Flow (Pdet at Qmax), and Maximum Detrusor Pressure (Pdet-max) except for pretreatment values of MCC and ∆_%_ MCC increase.

The baseline MCC in women with TC or CC genotypes was significantly lower than in TT homozygotes. However, there were no significant differences in MCC values and ∆_0–6_ MCC increases between the groups at six months after BoNT/A administration. Only the ∆_%_ MCC in women with at least one *ADRB3*:c.190 C allele was significantly higher than in women homozygous for the reference allele (TT). In addition, there were no significant differences between *ADRB3* TT homozygous patients and those carrying at least one C allele (TC heterozygotes or CC homozygotes) in terms of changes in OAB symptoms or scores from questionnaires ICIQ-OAB (parts A and B) and ICIQ-LUTS-QoL (parts A and B).

The *ADRB3*:rs4994 polymorphism with OAB treatment has been previously studied by several groups. In 2015, Gurocak et al. demonstrated that in Turkish children with overactive bladder, the inhibition of cholinergic activity with oxybutynin was efficient only in *ADRB3*:rs4994 TT homozygotes [23]. Another study, published by our group in 2022, included 115 Polish women with OAB in whom a response to BoNT/A was evaluated three months post-injection: the study showed no significant relationship between *ADRB3*:rs4994 polymorphism and treatment response as measured by the severity of OAB symptoms or changes in ICIQ-OAB and ICIQ-LUTS-QoL scores [27].

The pharmacogenetics of BoNT/A treatment has only been the subject of a few published studies [34–36]. The results of these studies have implied that primary resistance to BoNT/A is not associated with mutations in genes encoding synaptic vesicle glycoprotein 2 or synaptosome-associated protein 25 [34, 36]. However, a study conducted in 2019 demonstrated relationships between the rs3781719 polymorphism in the gene encoding calcitonin gene-related peptide 1 and the rs222749 polymorphism in the gene encoding transient receptor potential cation channel subfamily V member 1 and response to BoNT/A treatment in patients with chronic migraine [37].

A single administration of BoNT/A leads to a transient paralysis of human skeletal muscles that lasts longer than three months [26].

The profile of changes in the urodynamic parameters documented herein has demonstrated clearly that BoNT/A injection in women with OAB is an effective and safe method, improving the quality of life of the patients [38–40]. In 2007, Sahai et al. demonstrated that the beneficial effect of a single injection of BoNT/A (200 units) in patients with idiopathic OAB may persist for at least 24 weeks [41]. Nitti et al. stated that this substantially longer effect, than the circa four-month duration usually reported for skeletal muscle injections, suggests a target-tissue-dependent pharmacology for BoNT/A [40].

The present study has shown that treatment with 100 units of BoNT/A was equally effective in patients with iOAB and those with nOAB. Importantly, the pretreatment values of PVR and Pdet at Qmax in women with nOAB were significantly higher than in patients with iOAB. Notably, similar differences for Pdet at Qmax were also reported by Ghalayini and Al-Ghazo [42]. In a prospective study of BoNT/A treatment involving 14 patients with neurogenic detrusor overactivity and 16 patients with idiopathic detrusor overactivity, all resistant to anticholinergic treatment, Pdet at Qmax values in the first group were significantly higher than in the latter, but without a concomitant difference in PVR. It is also worth noting that, similarly to the present study, these authors found no significant differences between the groups for the pretreatment values of other urodynamic parameters, i.e., maximum cystometric capacity, maximum flow rate and maximum detrusor pressure [42].

Apart from pretreatment PVR and Pdet at Qmax, patients with iOAB did not differ from those with nOAB in terms of other baseline characteristics (Table 1), urodynamic parameters (Table 2), severity of OAB symptoms (Table 3) and ICIQ-OAB and ICIQ-LUTSqol questionnaire scores (Table 4). There were also no significant differences between iOAB and nOAB patients in the frequency distribution of both *ADRB3* genotypes or *ADRB3* alleles. This is why the main results given above concerning the relationship between treatment responses and *ADRB3* polymorphism were analyzed in a single pooled group of patients, using a logistic regression model with a correction for OAB type.

In 2008, Sahai et al. published the results of a prospective study involving 33 patients with OAB. The aim of the study was to verify whether baseline urodynamic parameters might predict urodynamic response to the injection of BoNT/A (200 units). Baseline MCC values in patients with a good response to BoNT/A injection were similar to those in poor responders. However, baseline values of Pdet-max in poor responders were significantly higher than in good responders. The authors of that study suggested that pretreatment Pdet-max > 110 cmH_2_O might be a predictor of poor response [28]. Our present study showed no significant differences in baseline Pdet-max values, either between patients with different OAB type (iOAB versus nOAB) or between patients with different *ADRB3* genotype (TT versus TC + CC). Additionally, it is worth emphasizing that baseline Pdet-max > 110 cmH_2_O was found in only 4 out of 124 TT homozygotes (3.2%) and 1 out of 14 women with at least one *ADRB3*:c.190 C allele (7.1%).

A principal limitation of the present study, precluding the formulation of any ultimate conclusions, is its relatively low statistical power. The latter results primarily from small sample size and low frequency of the *ADRB3* c.190 C allele in our patients with OAB. Using a free open-source software Open Epi (www.openepi.com) for epidemiological statistics, we have estimated that the minimum sample size should range from 1969 to 2668, with the number of women carrying at least once *ADRB3* C allele between 200 and 271. The frequency of the minor *ADRB3*:c.190 C allele in the present study (6.2%) was similar to that documented in our previously analyzed cohort (5.6%) [27]. It needs to be stressed that the values mentioned above are not only lower than the frequency of *ADRB3*:c.190 C alleles in Polish women without OAB (8.6–10.3%) [43–46], but also lower than in OAB patients from other European countries (8.1%, 9.0% and 10.8%) [20, 21, 47]. It is also worth noting that the distributions of *ADRB3*:rs4994 genotypes in our OAB patients, similarly to that, for example, in OAB Turkish patients [21], were not consistent with HWE. The HWE law, independently formulated in 1908 by Godfrey H. Hardy and by Wilhelm Weinberg, states that in a large random mating population at equilibrium (i.e., with no selection, migration or genetic drift), the biallelic genotype frequencies are functions of their allele frequencies. If the frequencies for allele A and allele a are p and q (= 1-p), then the frequencies for AA, Aa and aa genotypes are obtained from (p + q)^2^ = p^2^ + 2pq + q^2^, i.e., they are p^2^, 2pq, and q^2^, respectively [48, 49]. However, Namipashaki et al. underlined that HWE does not need to hold for a group of cases (e.g., OAB patients) since they are a non-random selection of individuals based on a phenotype of interest (e.g., disease) [50].

Finally, it is worth noting the limitations associated with urodynamic testing itself. Urodynamics is a “gold standard” and essential diagnostic tool for lower urinary tract dysfunction. However, the reliability, precision and accuracy of urodynamic tests can be disrupted by several pitfalls which depend on individual patients, on the physician or on the test per se as reviewed in 2020 by Finazzi Agrò et al. [51].

Taking into consideration both the results and limitations of our study, further research on the pharmacogenetics of BoNT/A in OAB patients should be based on genome-wide association studies in large groups of subjects and significant associations found in only one ethnic group should be verified in others. Subsequently, genetic variants identified in such studies must be assessed for linkage with genetic markers with actual functional significance [52].

In conclusion, the results of our present study do not preclude an association between the *ADRB3*:rs4994 polymorphism and urodynamic response to intra-detrusor injection of BoNT/A in patients with OAB.

## Data Availability

The datasets generated and/or analysed during the current study are available from the corresponding author upon reasonable request.

## Abbreviations

*ADRB3*: gene encoding beta-3 adrenergic receptor
BMI: Body Mass Index
BoNT/A: botulinum neurotoxin type A
bp: base pair(s)
χ^2^: Pearson’s chi-squared
COVID-19: Coronavirus Disease 2019
Δ _0−6_: absolute difference between pretreatment values of urodynamic parameters and respective values determined at six months post-injection
∆_%_: relative difference between pretreatment values of urodynamic parameters and respective values determined at six months post-injection
FSBF: First Sensation of Bladder Filling
HWE: Hardy-Weinberg Equilibrium
ICIQ-LUTS-QoL: International Consultation Incontinence Questionnaire-Lower Urinary Tract Symptoms-Quality of Life
ICQ-OAB: International Consultation on Incontinence Questionnaire-Overactive Bladder
ICS: International Continence Society (ICS)
iOAB: idiopathic overactive bladder
MCC: Maximum Cystometric Capacity
nOAB: neurogenic overactive bladder
OAB: overactive bladder
Pdet-max: Maximum Detrusor Pressure
Pdet at Qmax: Detrusor Pressure at Maximum Flow
PVR: Post-Void Residual
Qave: Average Flow Rate
Qmax: Maximum Flow Rate
